# Syphilis and Venereal Diseases

**Published:** 1877-02

**Authors:** 


### VI. Syphilis and Venereal Diseases.

1. *Gonorrhœal Balanitis.* Darvosky. (*Memorabilien,* 1876, No. 10.)

The inflammation of the prepuce, caused by gonorrhœal virus, produces a great œdema of the prepuce and a copious secretion from its mucous membrane. We are often warned, under such conditions, not to draw the prepuce back for fear of inducing paraphimosis. Dr. D., on the contrary, insists upon drawing back the prepuce in order to thoroughly cleanse the parts, and to apply the medicine directly upon the inflamed surface. After all the secretion is washed off, the whole everted surface of the prepuce is pencilled over with a solution of nitras argenti (thirty grains to the ounce); a small piece of linen saturated with the same lotion is then laid over the glans penis, and the prepuce drawn over it. During the first days the gray eschar produced by this cauterization is very quickly thrown off, wherefore the application of the nitras argenti should be repeated several times daily. Afterward, when the œdema of the prepuce has subsided and the discharge is greatly diminished, the eschar adheres for one day or longer, and the remedy must not be reapplied till the eschar has been thrown off.

2. *Syphilis of the Cochlea.* Roosa. (*N. Y. Med. Record,* 1876, No. 47.)

In his "Treatise on Diseases of the Ear," 1873, Dr. R. stated that he had yet to see a case of recovery from syphilitic disease of the labyrinth. Since that time he has seen several cases in which very great improvement has resulted from an antisyphilitic treatment, carried out faithfully and thoroughly. As to the diagnosis, the doctor's own remarks may be copied here:

1. Disease of the cochlea, as of the other parts of the labyrinth, usually, although not always, manifests itself suddenly. The patient can definitely fix upon a time when he became deaf, and when he began to have *tinnitus aurium.* This is true even when one side only is affected. The one-sided deafness would not be so quickly recognized were it not usually accompanied by tinnitus, vertigo, and often by unsteadiness of gait. Sudden loss of hearing and the sudden occurrence of tinnitus, vertigo, and staggering, are not, however, entirely peculiar to labyrinth disease, since it is well known that we sometimes, although rarely, have the same symptoms in cases of inspissated cerumen and catarrh of the middle ear. They are therefore only of pathognomonic value in connection with the objective examination and tests.

2. The tuning-fork is usually heard better or only in the sound or better ear. This would be a much more valuable test were the average powers of exact and objective examination better than we find them among our patients. The value of the tuning-fork is impaired by the willingness of even intelligent patients to believe that they hear all kinds of sounds, no matter how produced, better with the better ear. Then again, if both ears be affected, to nearly the same degree, the test has an extremely limited value.

3. The examination of the membrana tympani and the employment of the methods for inflating the middle ear, will usually give us reasonable conclusions as to the situation of a given disease of the ear, so that at the least we may exclude collections of fluid in the tympanic cavity in making a differential diagnosis between disease of the middle ear and of the labyrinth.

4. The piano, or any very similar musical instrument, will aid us in determining whether or not disease of the cochlea exists. The examination of cases that were unquestionably affections of the labyrinth shows that the power of appreciating low tones is the last to suffer, and the first to recover in most cases of disease of this part of the ear, so that these will be heard when the high ones are not heard at all, or if they are appreciated, they appear "*false*" or doubled. From our present knowledge of the physiology of hearing, when these symptoms are present, we must conclude that the cochlea is the seat of disease, even if it be secondarily affected.

5. The diagnosis of *syphilis* of the labyrinth depends in a great measure upon the same kind of evidence as that from which we conclude that a case of optic neuritis or choroiditis is syphilitic; that is to say, the history and the presence of other symptoms as eruption, mucous patches, etc. It should not be forgotten, however, that the occurrence of labyrinth disease, in a person who has probably had the initial lesion of syphilis, even if no other symptoms are present, is a very suspicious circumstance, which should lead to a careful weighing of the indications for and against a mercurial treatment.

